# Propagation Regimes and Signal Enhancement Mechanisms of Collinear Double-Pulse Plasma with Varying Inter-Pulse Delays

**DOI:** 10.3390/s25113409

**Published:** 2025-05-28

**Authors:** Yang Zhao, Lei Zhang, Zhihui Tian, Xiuqing Zhang, Jiandong Bai, Wangbao Yin

**Affiliations:** 1 School of Semiconductor and Physics, North University of China, Taiyuan 030051, China; yangzhao@nuc.edu.cn (Y.Z.); zhangxiuqing@nuc.edu.cn (X.Z.); jdbai@nuc.edu.cn (J.B.); 2 State Key Laboratory of Quantum Optics Technologies and Devices, Institute of Laser Spectroscopy, Shanxi University, Taiyuan 030006, China; ywb65@sxu.edu.cn; 3 Collaborative Innovation Center of Extreme Optics, Shanxi University, Taiyuan 030006, China; 4 School of Physics and Electronic Information Engineering, Hubei Engineering University, Xiaogan 432000, China; tianzh@hbeu.edu.cn

**Keywords:** collinear double-pulse laser-induced plasma, collinear double-pulse laser-induced breakdown spectroscopy (DP-LIBS), laser-supported absorption (LSA) wave, signal enhancement mechanisms

## Abstract

Laser-induced breakdown spectroscopy (LIBS) is an in situ analytical technique. Compared to traditional single-pulse LIBS (SP-LIBS), collinear double-pulse LIBS (DP-LIBS) is a promising technique due to its lower limit of detection for trace elements. In this paper, we analyze the spectral and image information obtained from the emissions emitted by single/double pulse (SP/DP) laser-induced plasmas. The types of laser-supported absorption (LSA) waves of the plasmas were determined according to the interactions among the ablation vapor, the ambient gas, and the laser. Furthermore, the influence mechanisms of plasma shielding on DP-LIBS signal intensity enhancement with different inter-pulse delay were investigated. In our experimental conditions, the propagation regime of SP plasma is a laser-supported combustion (LSC) wave. The DP plasmas with short inter-pulse delays show the characteristics of a laser-supported detonation (LSD) wave, and the enhancement mechanism is mainly reheating for pre-plasma. On the contrary, the DP plasmas with longer inter-pulse delays show the characteristics of a LSC wave, and the increase in laser ablation is a major contributing factor to the signal improvement. In addition, the spectral lines, which are difficult to excite by SP-LIBS, can be obtained by selecting an appropriate inter-pulse delay and setting a short delay, which provides a new idea for the measurement of trace elements.

## 1. Introduction

Laser-induced breakdown spectroscopy (LIBS) is a developing analytical technique. A high-energy laser is used to ablate minute quantities of materials from the target, and the spectra emitted from the resulting high-temperature plasmas are analyzed to obtain the elemental composition of the target. LIBS is particularly suitable for industrial process monitoring and product quality control due to the advantages of high speed, little or no sample preparation, and multi-element analysis [1,2,3,4]. However, the detection accuracy and sensitivity of LIBS for trace elements have not yet met the requirements of certain industries, necessitating further advancements [5,6].

In recent years, many researchers have devoted a lot of energy to this issue and proposed a variety of signal enhancement schemes, such as double-pulse (DP) excitation, picosecond and femtosecond pulse excitation, spark discharge-assisted excitation, microwave-assisted excitation, spatial confinement, and magnetic confinement [7,8,9,10]. Among these methods, DP-LIBS has emerged as a highly effective approach, as it uses two laser pulses (with inter-pulse delays ranging from hundreds of nanoseconds to a few microseconds) to more efficiently deliver laser energy to the target and plasma. DP-LIBS is a hotspot research area due to some of its merits, including an improved signal-to-noise ratio [11], increases in signal intensity by factors of two to thirty-three [12,13,14,15], reductions in the limit of detection (LOD) by almost tenfold [16,17,18], and substantial enhancements in quantitative analysis performance [13].

The generally acknowledged interpretations of the enhancement mechanisms of DP-LIBS are the following: 1. Due to the plasma shielding, the pre-plasma generated by the pre-pulse absorbs the energy from the reheating pulse. 2. The shockwave generated by the pre-pulse reduces ambient gas density, creating a low-pressure region at the target surface. Following the second excitation, the reheated plasma expands rapidly in this rarefied region according to fluid dynamics, resulting in a larger plume volume that reduces plasma shielding and increases ablation mass [19,20,21,22]. Additionally, Pasquini et al. [23] pointed out that, in addition to decreasing the breakdown threshold, effective laser–plasma coupling is also a crucial factor for enhancing DP-LIBS performance. Kepes et al. [24] found that reheating during ablation significantly improves the spatial homogeneity of the plasma temperature and electron density.

In general, most DP-LIBS research primarily uses larger signal acquisition delays and gate widths (>500 ns) [25]. However, understanding the structure and morphology of plasmas in the early stage is essential for comprehending their entire propagation and evolution. Furthermore, only a limited number of studies have combined the propagation regimes of laser-supported absorption (LSA) waves and species distribution to elucidate the enhancement mechanisms of DP-LIBS. The propagation mechanisms of plasma can be divided into two primary regimes: laser-supported combustion (LSC) waves and laser-supported detonation (LSD) waves. This classification depends on whether the laser energy is mainly absorbed by vapor or shocked gas. The shielding effect of plasma for LSD waves exists in the shock gas at the forefront of plasma, where the plasma exhibits a mixed structure. For LSC waves, the shielding effect occurs in the ablation vapor at the center of the plasma, resulting in a layered structure of plasma.

Based on the propagation direction and the temporal sequence of the laser pulses, the DP-LIBS can be categorized into three types: collinear, orthogonal pre-ablation, and orthogonal reheating. Among these schemes, the collinear DP-LIBS is more suitable for industrial applications due to its simple configurations and adjustability. In this study, the emission spectra and species emissivity images of collinear SP/DP plasmas with short delay times and narrow gates were obtained, and the interactions between vapor and ambient gases were analyzed. The purpose is to elucidate the propagation characteristics of the plasma and investigate the physical principles and signal enhancement mechanisms of collinear DP-LIBS. A novel idea is proposed to decrease the limit of detection for trace elements while providing theoretical and technological support for the application of DP-LIBS in online quality monitoring of industrial processes.

## 2. Experimental Setup

**Figure 1:**
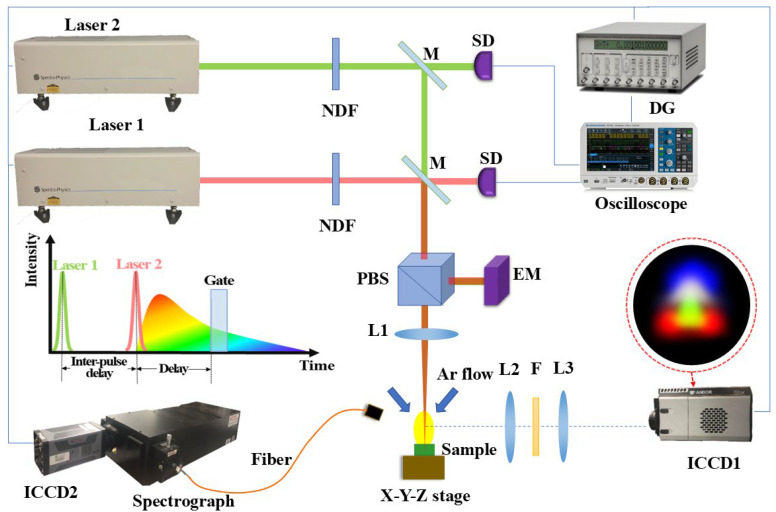
Schematic of the experimental DP-LIBS setup. Components include NDF (neutral density filters), M (mirror), PBS (polarizing beam splitter), EM (energy meter), DG (digital delay generator), SD (silicon detectors), ICCD (intensified CCD), L1, L2, L3, and L4 (lenses), and F (bandpass filter).

The DP-LIBS experimental setup, as shown in Figure 1, mainly consisted of three parts: the plasma excitation system, the dual-wavelength differential spectroscopic imaging system, and the spectra acquisition system.

The excitation source for DP-LIBS was two high-energy pulsed lasers (Sunnyvale, CA, USA, Spectra Physics, INDIHG-20, wavelength: 1064 nm, pulse duration: 7 ns, and repetition frequency: 20 Hz). The digital delay generator (Sunnyvale, CA, USA, Stanford Research System, DG535) was used to synchronize two lasers and adjust inter-pulse delays, and the actual inter-pulse delays were monitored by two silicon detectors (Newton, NJ, USA, Thorlabs, DET10A/M) in real time. The neutral density filters (NDF) were used to adjust the laser energy to ensure that the pulse spots were evenly distributed. In order to monitor the incident laser energy, the fraction of the laser reflected by the polarizing beam splitter (PBS) was measured using an energy meter (Jerusalem, Israel, Ophir, Nova II) that was perfectly calibrated. A lens (L1) with a focal length of 50 mm was used for focusing the laser beams below the sample surface, and the spot size was about 0.6 mm. The sample was mounted on an X-Y-Z stage so that each laser pulse hit a different spot. To make certain that the plasma expanded in pure argon at atmospheric pressure, two gas tubes were positioned above the sample surface, and the argon continually blew close to the laser focus. This excitation system was capable of emitting laser in two modes. The single-pulse mode used only laser 1 with an irradiance of 2 GW/cm^2^. The collinear dual-pulse mode utilized laser 1 and laser 2 with irradiances of 1 GW/cm^2^ and 1 GW/cm^2^, respectively. The timing and sequence of the laser pulses are illustrated in Figure 1, with inter-pulse delays between the two pulses ranging from 200 ns to 15000 ns.

The dual-wavelength differential spectroscopic imaging system was mounted on the right side of the plasma and perpendicular to the laser incident axis. Please refer to the published papers for more detailed information [26,27]. In summary, an ICCD1 camera (Belfast, United Kingdom, Andor, iStar DH334T-18U-03) was used to obtain the images of a certain species distribution in plasma through a 4F optical system composed of two lenses (L2 and L3) and a pair of bandpass filters (F1 and F2) with bandwidths of 6–10 nm. The center wavelength of F1 corresponded to the chosen emission line of the species, while the center wavelength of F2 was closely related to F1, and there were no lines in F2’s passband. The ICCD1 recorded emission intensity images of the species through F1 while recording the continuous background using F2. In order to improve the signal-to-noise ratio and reduce the fluctuation, the specific ICCD gains (see Table 1) were set for each species, and each image accumulated 60 plasmas. The obtained images were processed by transmittance correction, continuous background subtraction, and Abel inversion. Finally, the emissivity images of the species were obtained. This system can visually display the evolution process of various species in the plasma and directly obtain the morphology and structure of the plasma.

The spectra acquisition system was positioned on the left side of the plasma. The fluorescence was transmitted to the grating spectrograph (Trenton, NJ, USA, Princeton Instruments, SP-2750) equipped with ICCD2 (Trenton, NJ, USA, Princeton Instruments, PI-MAX4-1024i) through an all-silica optical fiber at a 45-degree angle. The time-resolved spectra can be obtained by adjusting the delay and gate width of the ICCD2. A low-pressure Hg lamp (Irvine, CA, USA, Newport, 6048) calibrated the spectrograph’s wavelength, while a deuterium-halogen light source (Apeldoorn, Netherlands, Avantes, AvaLightDH-S-BAL) calibrated the intensity of spectra.

The materials used in this experiment were pure aluminum (≥99.99%), with the surface polished using sandpaper before each measurement set. The spectral lines and center wavelengths of the filters used for imaging are listed in Table 1. Among them, the Al II line is a superposition of 15 lines, and Al II 358.66 nm listed in Table 1 is the one with the highest transition probability.

## 3. Results

### 3.1. Comparison of SP and DP LIBS Spectra

#### 3.1.1. Effect of Spectral Acquisition Delays on Spectra

**Figure 2:**
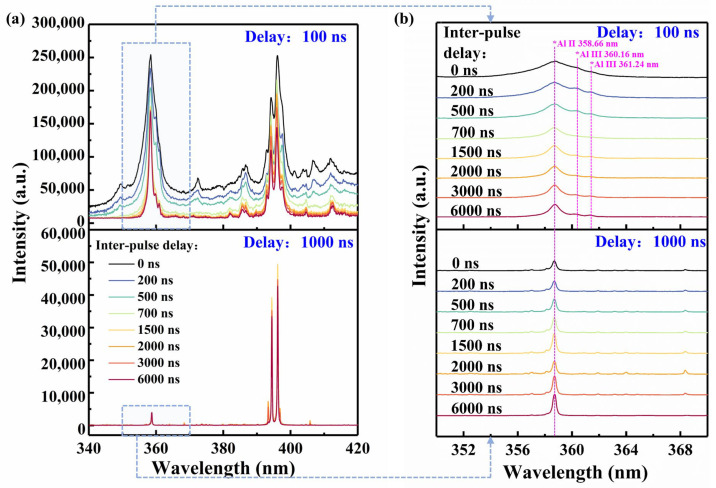
(**a**) Emission spectra of SP aluminum plasma (horizontal coordinate of zero) and DP aluminum plasma (inter-pulse delays of 200 ns, 500 ns, 700 ns, 1500 ns, 2000 ns, 3000 ns, and 6000 ns) in Ar ambient gas at delays of 100 ns and 1000 ns, corresponding to gate widths of 5 ns and 500 ns. (**b**) A magnified view of (**a**) in the range of 350–370 nm.

For DP-LIBS, only a few experiments have been conducted with short delay times and narrow gates. As a result, the spectral acquisition delays and gate widths of ICCD2 were set to 100 ns and 5 ns, respectively, in addition to the typical 1000 ns and 500 ns. Under the above acquisition conditions, the emission spectra of SP Al plasma (horizontal coordinate of zero) and DP Al plasma (inter-pulse delay 200 ns, 500 ns, 700 ns, 1500 ns, 2000 ns, 3000 ns, and 6000 ns) in the range of 340–420 nm are shown in Figure 2a. Figure 2b shows a magnified view of Figure 2a in the range of 350–370 nm, and the stacked lines are separated for clarity, so there is no vertical scale. Comparing the spectra at delays of 100 ns and 1000 ns, it can be found that the continuum background at 100 ns is significantly stronger, resulting in wider full width at half maximums (FWHMs) of the spectral lines. Notably, the spectral lines of doubly ionized Al species—Al III at 360.16 nm and 361.24 nm—are detectable at 100 ns, regardless of the method (SP or DP).

**Figure 3:**
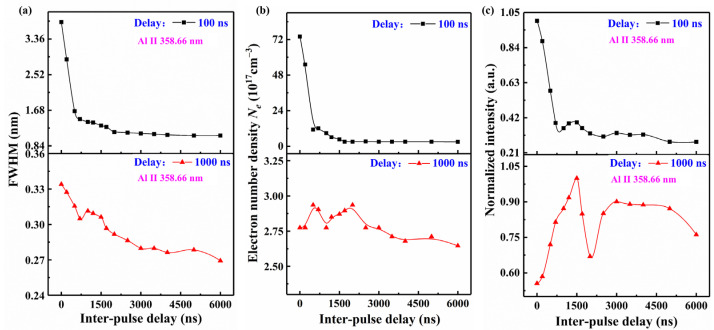
(**a**) FWHM of the Al II 358.66 nm spectral line emitted from SP and DP plasmas at inter-pulse delays of 100 ns (gate width: 5 ns) and 1000 ns (gate width: 500 ns) in an argon ambient gas environment. (**b**) *Ne* as a function of inter-pulse delay under the same experimental conditions. (**c**) Normalized signal intensity of Al II 358.66 nm plotted against inter-pulse delays, highlighting trends for SP and DP plasmas at 100 ns and 1000 ns acquisition delays.

The electron number density *Ne* is the cause of the aforementioned phenomenon. The *Ne* was calculated by [28]

(1) $$Ne\left({cm}^{-3}\right)=10^{17}{\left[{\Delta \lambda }_{1/2}(nm)/0.549\right]}^{1.4713}$$

where ${\Delta \lambda }_{1/2}$ represents the FWHM of the Stark-broadened Hα line at 656.28 nm. The curves of *Ne* as a function of inter-pulse delays at 100 ns and 1000 ns are shown in Figure 3b. It can be seen that the *Ne* of the initial stage of plasma expansion is high due to a stronger continuous background brought on by Bremsstrahlung. This increased ionization allows the detection of the doubly ionized lines, although these lines have a very short lifetime and are not detectable at 200–300 ns.

#### 3.1.2. Effect of Inter-Pulse Delays on Spectra

The spectra of SP and DP plasmas with varying inter-pulse delays were compared at the same delay. Compared to DP-LIBS, the spectra of SP-LIBS show the highest continuous background at a delay of 100 ns. The FWHM of Al II 358.66 nm is 3.76 nm for SP-LIBS. In contrast, the Al II 358.66 nm of DP-LIBS has a lower FWHM of 1.08–2.88 nm, and decreases with the inter-pulse delay. At a delay of 1000 ns, both SP and DP plasmas show a similarly low level of continuous background, and the FWHM of Al II 358.66 nm fluctuates within a narrow range of 0.27 to 0.33 nm. By comparing the intensity of spectral lines emitted from SP and DP plasma, we found maximum enhancement factors of 1.8 for Al II 358.66 nm, 1.6 for Al I 396.15 nm, and 1.4 for Al I 394.40 nm.

The effect of the inter-pulse delays on the continuous background and FWHM of the spectral lines is primarily due to the short inter-pulse delay, during which the second pulse interacts with the pre-plasma characterized by a high *Ne*. A portion of the energy from the second pulse is absorbed by free electrons, further increasing *Ne* through collisions between electrons and neutral or singly ionized atoms. Consequently, the *Ne* of plasma with the shorter inter-pulse delay is higher at 100 ns, as well as having a larger continuous background and wider FWHM. However, as the inter-pulse delays exceed 2000 ns, these influences decrease significantly. By contrast, the *Ne* drops to the same level at the delay of 1000 ns, resulting in minimal variation in the continuum background.

Figure 3c depicts the variation in the normalized intensity of Al II 358.66 nm as a function of inter-pulse delay. The intensity of Al II emitted from the SP plasma (with an inter-pulse delay of 0 ns) is notably lower than that emitted from the DP plasma at 1000 ns. However, the opposite trend is observed at 100 ns due to the larger continuous background in the SP plasma. The influence of the inter-pulse delays of DP plasma on the intensity of Al II was further investigated. At a 100 ns delay, the intensity of Al II for inter-pulse delays ranging from 100 to 700 ns is also significantly affected by the larger continuous background. After excluding these data points, the trends of the curves at delays of 100 ns and 1000 ns become consistent, both showing two peaks at inter-pulse delays of 1500 ns and 3000 ns. The underlying mechanisms for these observations will be discussed in Section 3.4, in conjunction with plasma imaging analysis. It is notable that the trends of the FWHM and normalized intensity of other Al II lines (e.g., Al II 466.31 nm) are similar to those of Al II 358.66 nm. The results derived from our analysis of Al II 358.66 nm can be extended to other Al II lines.

**Figure 4:**
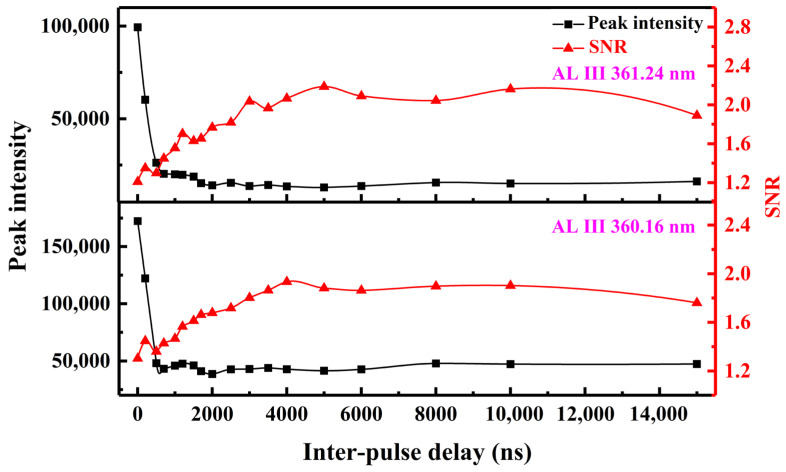
Peak intensity and signal to noise ratio (SNR) of the Al III 360.16 nm and 361.24 nm spectral lines as a function of inter-pulse delays.

The effect of inter-pulse delays on the Al III 360.16 nm and 361.24 nm spectral lines is illustrated in Figure 4. Due to the strong continuous background of SP plasma, the signal-to-noise ratios (SNRs) of these doubly ionized lines of SP-LIBS are very low. However, in DP-LIBS, the SNRs improve significantly as the inter-pulse delay increases to 4000–5000 ns. In summary, DP-LIBS demonstrates the ability to excite spectral lines—such as highly ionized lines and trace element lines—that are typically inaccessible using conventional SP-LIBS. The aforementioned characteristics of DP-LIBS will offer a novel LIBS scheme for measuring trace elements: selecting a suitable inter-pulse delay to excite the plasma and setting a short delay time for spectral acquisition. The spectra with a low continuum background and a strong intensity of the spectral lines can be measured.

### 3.2. Comparison of Space-Time Evolution of SP and DP Plasma

#### 3.2.1. Morphology and Structure of SP Plasma

**Figure 5:**
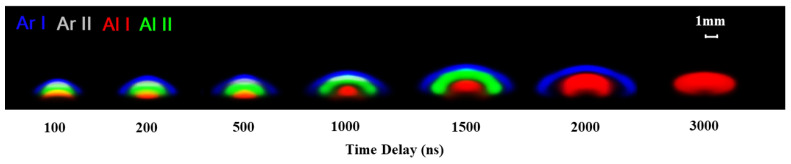
Normalized emissivity images of various species within the SP plasma at different delays. The species are represented by different colors: blue for argon atoms (Ar I), gray for argon ions (Ar II), red for aluminum atoms (Al I), and green for aluminum ions (Al II). The delay times investigated include 100 ns, 200 ns, 500 ns, 1000 ns, 1500 ns, 2000 ns, and 3000 ns, with corresponding gate widths of 5 ns, 7 ns, 10 ns, 50 ns, 100 ns, 150 ns, and 200 ns, respectively.

As shown in Figure 5, the dual-wavelength differential spectroscopy was used to obtain emissivity images of species with different colors: blue for argon atoms (Ar I), gray for argon ions (Ar II), red for aluminum atoms (Al I), and green for aluminum ions (Al II). To better represent the internal structure of the plasma, each emissivity image was normalized by its maximum value.

In this ablation mechanism, the plasma including all emitted species exhibits a hemispherical shape and a distinct layer structure, with the order of argon atoms, argon ions, aluminum ions, and aluminum atoms along the laser incidence direction (i.e., the symmetry axis of the plasma in the figure from top to bottom). The layer of argon ions covers the vapor plume, composed of aluminum ions and atoms, while the outermost layer of argon atoms envelopes the entire plume.

The ordered arrangement of species layer indicates a region of highest temperature exists between the excited argon atom layer and the front of the aluminum vapor. The global expansion and cooling of the aluminum vapor is more determined by fluid dynamics for the longer delay. The thin layer of argon ions dissipates after 1000 ns, and the aluminum ions vanish after 1500 ns.

**Figure 6:**
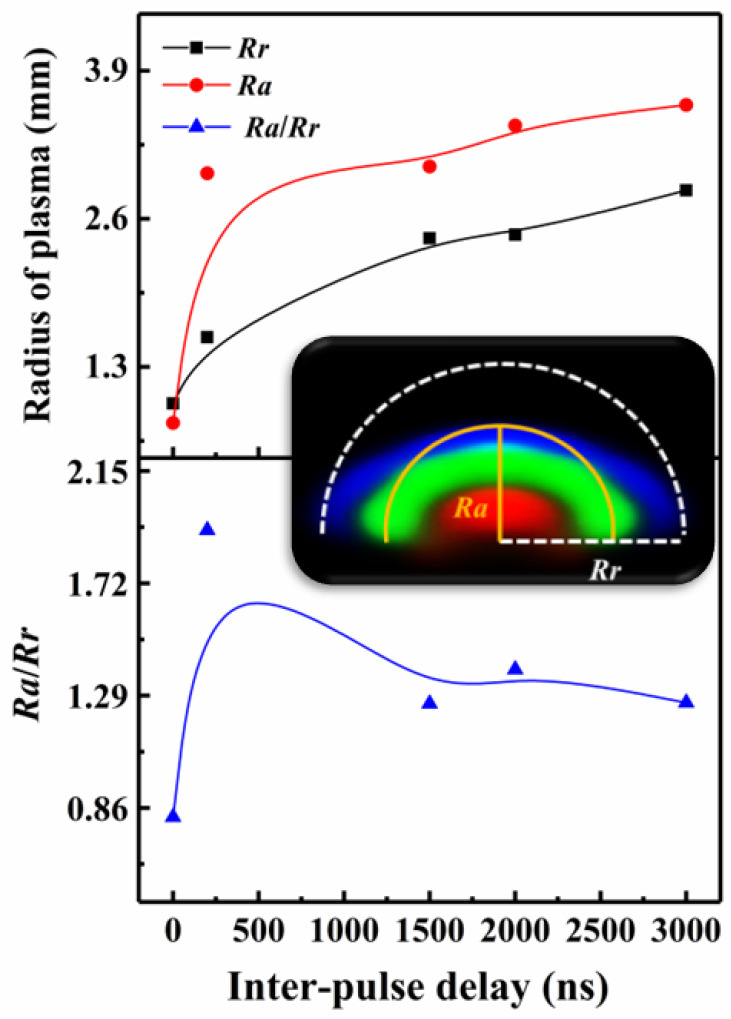
The axial expansion radius *Ra*, radial expansion radius *Rr*, and the ratio of *Ra* to *Rr* for SP plasma (horizontal coordinate of zero) and DP plasma (inter-pulse delay 200 ns, 1500 ns, 2000 ns, and 3000 ns) in argon ambient gas environment. The measurements were taken at a delay of 100 ns with a gate width of 5 ns.

As shown in Figure 6, the expansion radii of the plasma were measured by plotting two concentric semicircles. The centers of both semicircles aligned with the center of the laser spot, which corresponded to the location of the plasma center on the sample surface. *Ra* is the axial radius of the plasma, which accounts for its axial expansion, while *Rr* represents half of the maximum radial expansion range, reflecting radial expansion of the plasma. The *Ra* and *Rr* of SP and DP plasma, alongside the ratio of *Ra* to *Rr* are displayed at a delay of 100 ns in Figure 6. The representative values for analyzing the axial and radial expansion of plasmas are provided under different laser excitation conditions. For SP Al (horizontal coordinate of zero) at 100 ns, the measured values are *Ra* = 0.807 mm, *Rr* = 0.978 mm, and the ratio of *Ra* to *Rr* is 0.83. These results indicate that the SP plasma expands more quickly in the radial direction.

#### 3.2.2. Morphology and Structure of DP Plasma

**Figure 7:**
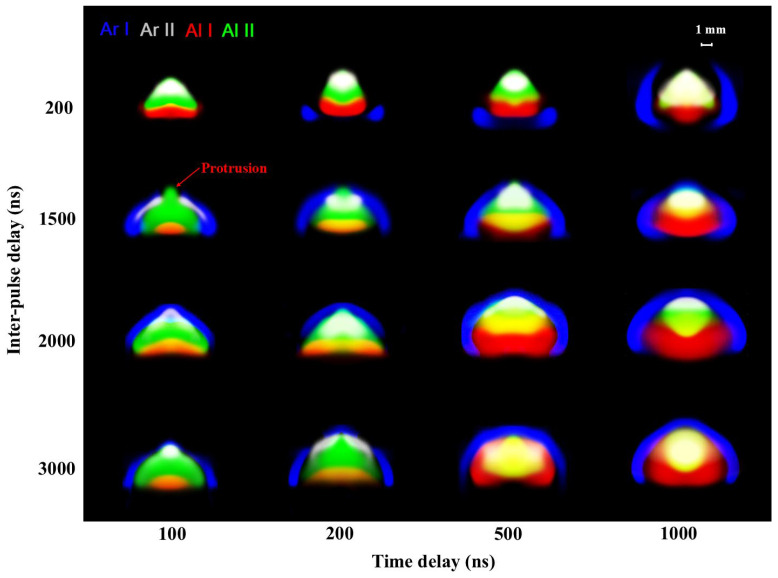
Emissivity images of species in DP plasma at inter-pulse delays of 200 ns, 1500 ns, 2000 ns, and 3000 ns. The species are represented by different colors: blue for argon atoms (Ar I), gray for argon ions (Ar II), red for aluminum atoms (Al I), and green for aluminum ions (Al II). The images were obtained at delay times of 100 ns, 200 ns, 500 ns, 1000 ns, 1500 ns, 2000 ns, and 3000 ns, with corresponding gate widths of 5 ns, 7 ns, 10 ns, 50 ns, 100 ns, 150 ns, and 200 ns, respectively.

The emissivity images of DP plasma with the inter-pulse delays of 200 ns, 1500 ns, 2000 ns, and 3000 ns (selected from Figure 3c) are shown in Figure 7. Similar to the SP plasma, the DP plasma exhibits a hemispherical shape. However, as shown in Figure 6, the *Ra* of the DP plasma can reach up to 4.5 times larger than that of the SP plasma, indicating significantly enhanced axial expansion. Both the *Ra* and *Rr* of the DP plasma increase with the inter-pulse delays, whereas the ratios of *Ra* to *Rr* decrease. The *Ra* is larger than the *Rr* for the DP plasma. Therefore, the DP plasma expands more quickly in the axial direction.

There are differences in the internal structure of the DP plasma with different inter-pulse delays. The first row of Figure 7 shows the emissivity images of DP plasma with a short inter-pulse delay of 200 ns. The argon atoms are completely ionized at the delay of 100 ns, and the argon ions overlap with the aluminum ions (creating a bright white region in Figure 7 due to the mixture of gray and green). This overlap results in the formation of a thick mixed zone, located behind the shockwave front. This structure indicates that the mixed thick zone with a high temperature is directly in contact with the undisturbed ambient gas, and the argon ions persist for a prolonged period. As the delay increases to 200 ns, the argon atoms are generated from a region near the target surface and gradually envelope the plume core.

The internal structure of DP plasma with inter-pulse delays of 1500 ns, 2000 ns, and 3000 ns is similar to that of SP plasma, exhibiting an obvious layer structure. In these cases, the argon ion layer and aluminum ion layer do not overlap or have only a small overlap region. However, a notable difference appears for DP plasma with the inter-pulse delay of 1500 ns: the axial expansion velocity of aluminum ions exceeds that of argon ions at the delay of 100 ns, causing the aluminum ions to breach the layer of argon ions and form a small “protrusion” on its originally hemispherical shape. The detailed explanation for this phenomenon will be provided in Section 3.4, combined with the propagation types of SP and DP plasma.

### 3.3. Propagation Types of SP and DP Plasma

In references [29,30,31] and our previous works [26,27,32], the experimentally observed propagation regimes of SP and DP plasmas were discussed, and the results are summarized in Table 2. SP plasma exhibits a distinct layered structure, and the degree of ionization in the shock gas layer is relatively low. Because of the low density and temperature of argon, the laser penetrates through the shocked gas layer and deposits on the ablation vapor plume. The excitation and ionization of argon are not directly caused by laser energy absorption, but by the interaction between argon and the heated vapor plasma core, such as compression, heat conduction, and radiation transfer. Due to the low density and temperature of argon, the laser penetrates through the shocked gas layer and deposits energy in the ablation vapor plume. Therefore, the laser absorption region is located in the middle of the plasma. These characteristics of SP plasma are in accordance with LSC wave.

For DP plasma with an inter-pulse delay of 200 ns, the shocked gas layer is widely distributed and exhibits a high degree of ionization. This is because the shocked gas layer of pre-plasma has high density and temperature during the brief expansion time (200 ns). There is strong coupling between the second laser pulse (laser 2) and the shocked gas layer of pre-plasma, leading to the direct absorption of a significant amount of laser energy. At this stage, the shocked gas layer not only propagates forward but also recoils backward, forming a mixed region with the ablation vapor. The laser-absorbing zone, which is located at the top of the plasma, is the driving force of the expansion process in the direction of the laser incidence, and the plasma appears elongated for this reason. In summary, the plasma propagation regime belongs to the LSD wave.

The internal layered structures of DP plasmas with longer inter-pulse delays (1500 ns, 2000 ns, and 3000 ns) are similar to those of SP plasma. This similarity arises because the prolonged expansion time of the pre-plasma reduces the density of the shocked gas layer. As a result, the ablation vapor becomes the primary region for laser energy absorption again, exhibiting characteristics consistent with the LSC wave propagation regime.

### 3.4. Signal Enhancement Mechanisms of DP-LIBS

The signal enhancement of the DP-LIBS can be attributed to two primary reasons: 1. due to the plasma shielding effect, the pre-plasma absorbs the energy of the second laser pulse (laser 2), leading to a re-heating process of the plasma; 2. the first laser (laser 1) improved environmental conditions for the laser 2. According to shadowgraph studies [33,34], the ambient gas is compressed into a thin layer behind the shockwave front after the initial plasma generation. The internal pressure in the plume decreases as the shockwave expands outwards from the target, creating more diluted conditions for the plasma induced by laser 2. Additionally, a stronger coupling between the laser 2 and the target leads to a higher ablation mass of material.

Referring to the intensity of the Al II line at a delay of 1000 ns (shown in Figure 3c), the reheating effect is more effective for shorter inter-pulse delays (200 ns and 1500 ns) due to the higher density of the pre-plasma. Consequently, the pre-plasma reheating is the main enhancement mechanism.

Specifically, for the inter-pulse delay of 200 ns, the diminished signal enhancement of the Al II line is owing to the strong coupling between laser 2 and the shocked gas layer of the pre-plasma. In contrast, for the inter-pulse delay of 1500 ns, the axial expansion speed of aluminum ions exceeds that of argon ions, indicating a stronger interaction between laser 2 and the ablation vapor of the pre-plasma. The argon ion layer is disrupted by the aluminum ions, forming a “protrusion” in the emissivity images, and the Al II line is greatly intensified as a result.

As the inter-pulse delay increases to 2000 ns, the enhancement mechanism remains dominated by pre-plasma reheating. However, a decrease in pre-plasma density reduces the reheating efficiency of ablation vapor and limits the intensity enhancement of the Al II line, leading to a dip for 2000 ns as observed in Figure 3c.

With an even longer inter-pulse delay (3000 ns), the pre-plasma density further decreases, reducing its shielding effect on laser 2. The extremely rarefied ambient conditions enhance the laser–target interaction, making the increase in ablation mass the primary enhancement mechanism. As shown in Figure 3c, the intensity of the Al II line emitted from DP plasma with a 1500 ns inter-pulse delay is stronger than that with a 3000 ns inter-pulse delay. Thus, as an enhancement mechanism, plasma reheating is more effective than the increase in the ablation mass under the experimental conditions. The enhancement mechanisms for collinear DP-LIBS with different pulse delays are also summarized in Table 2.

## 4. Conclusions

In this study, a dual-wavelength differential spectroscopic imaging system and a time-resolved spectra acquisition system were employed to obtain both spectral and image information from SP and collinear DP aluminum plasmas with varying inter-pulse delays. The expansion dynamics of the vapor in the ambient gas and the relative distribution of different species were analyzed. Additionally, we investigated the propagation regimes of the plasmas and the signal enhancement mechanisms of DP-LIBS.

The results indicate that DP-LIBS can effectively enhance spectral quality compared to conventional SP-LIBS. The maximum enhancement factors of Al II 358.66 nm, Al I 396.15 nm and Al I 394.40 nm are 1.8, 1.6, and 1.4, respectively. The doubly-ionized lines of Al species can be emitted in the earliest stages of plasma lifetime. Under the experimental conditions in this research, the SP plasma exhibits an obvious layered structure with a lower ionization degree in the shocked gas layer. The characteristics indicate the propagation regime is an LSC wave. In contrast, DP plasmas with short inter-pulse delays exhibit a high ionization degree in the shocked gas layer, with an overlapping distribution of argon and aluminum ions observed at the plasma’s top due to strong coupling between the second pulse and the shocked gas layer. In this condition, the propagation regime belongs to the LSD wave, and the signal enhancement mechanism relies on the high reheating efficiency of pre-plasma. For larger inter-pulse delays, internal layered structures of DP plasma are similar to those observed in SP plasma. This similarity arises because the longer expansion time of the pre-plasma reduces the density of the shocked gas layer and reduces the shielding effect. As a result, ablation vapor becomes the main area for laser energy absorption, presenting characteristics of LSC waves. At this time, the rarefaction within the shockwave can enhance coupling between the second pulse and the target, with the dominant enhancement mechanism being an increase in ablation mass.

The spectral lines that are difficult to excite using conventional SP-LIBS can be obtained using DP-LIBS by selecting an appropriate inter-pulse delay for the two pulses and setting a short delay time and narrow gate as acquisition parameters. This approach provides a new perspective for trace element measurement in LIBS, and we look forward to further technological breakthroughs.

In addition to DP-LIBS, several potential strategies exist for improving the analytical performance of LIBS in practical industrial applications. For example, by combining LIBS with high-reproducibility and high-sensitivity analytical techniques such as laser ablation inductively coupled plasma mass spectrometry (LA-ICP-MS), X-ray fluorescence spectrometry (XRF), and laser-induced fluorescence (LIF), the comprehensive quantitative analytical capabilities for multiple elements can be significantly enhanced while retaining the advantage of the broad elemental dynamic range of LIBS. Furthermore, introducing advanced AI-based chemometric algorithms to optimize quantitative models also provides an effective approach for improving trace element detection accuracy.

## Figures and Tables

**Table 1:** The filter pairs used for the observation of Al and Ar atomic and ionic species. Additionally included are the ICCD gains for each species under different laser excitation modes.

Species	Emission Line (nm)	Center Wavelength of Filter F1 (nm)	Center Wavelength of Filter F2 (nm)	ICCD Gain	
				SP Plasma	DP Plasma
Al I	394.40	396	365	300	200
	396.15				
Al II	358.66	358	365	300	200
Ar I	763.51	764	786	500	300
Ar II	484.78	488	530	500	300

**Table 2:** Propagation regimes and enhancement mechanisms of SP plasma and DP plasma with different inter-pulse delays.

	SP Plasma	DP Plasma with Different Inter-Pulse Delays			
		200 ns	1500 ns	2000 ns	3000 ns
Propagation regime	LSC wave	LSD wave	LSC wave	LSC wave	LSC wave
Enhancement mechanism	-	Reheating	Reheating	Reheating	Increase in ablation mass

## Data Availability

The raw data supporting the conclusions of this article will be made available by the authors on request.

## Abbreviations

The following abbreviations are used in this manuscript:

LIBS	Laser-induced breakdown spectroscopy
SP-LIBS	Single-pulse laser-induced breakdown spectroscopy
DP-LIBS	Double-pulse laser-induced breakdown spectroscopy
SP	Single pulse
DP	Double pulse
LSA wave	Laser-supported absorption wave
LSC wave	Laser-supported combustion wave
LSD wave	Laser-supported detonation wave
LOD	Limit of detection
FWHM	Full width at half maximum
LA-ICP-MS	Laser ablation inductively coupled plasma mass spectrometry
XRF	X-ray fluorescence spectrometry
LIF	Laser-induced fluorescence
